# Dietary Protein Affects the Growth Response and Tissue Composition of Juvenile Slipper Lobster (*Thenus australiensis*)

**DOI:** 10.3390/ani14233363

**Published:** 2024-11-22

**Authors:** Andrea Williamson, Chris G. Carter, M. Basseer Codabaccus, Quinn P. Fitzgibbon, Gregory G. Smith

**Affiliations:** Institute for Marine and Antarctic Studies (IMAS), University of Tasmania, Private Bag 49, Hobart, TAS 7001, Australia

**Keywords:** slipper lobster, protein, energy, digestibility, feed development

## Abstract

The effect of different dietary protein levels on apparent feed intake, apparent digestibility, body composition, nutrient retention, and growth efficiency of juvenile slipper lobster (*Thenus australiensis*) were evaluated for 74 days. Three isoenergetic and isolipidic feeds were formulated with crude protein levels of 445, 490, and 551 g kg^−1^ DM, designated as P45, P50, and P55, respectively. Lobsters were fed one of these feeds in quadruplicate tanks, with eight lobsters (mean initial wet weight 6.1 ± 0.3 g) per tank in a recirculating seawater system at 27 °C. Increasing dietary protein levels significantly increased apparent feed intake, final dry weight, and daily weight gain. Dietary protein affected whole-body protein, lipid, and energy content. Lobsters fed P55 had significantly higher lipid and energy content than those fed P50 and P45. Protein content was significantly higher in lobsters fed P55 than P50, while P45 was not significantly different from either P50 or P55. Slipper lobsters grew best on a feed containing 550 g kg^−1^ crude protein (equivalent to 518 g kg^−1^ digestible protein), providing initial insights into their protein dietary requirement. This study established a foundation for developing standardized feeds, with further research needed to optimize growth performance and nutrient utilization.

## Simple Summary

The slipper lobster is being studied because it is valuable commercially and is a good candidate for intensive aquaculture. Recently, researchers successfully raised these lobsters in captivity using only formulated feeds. This study aimed to understand the protein needs of juvenile slipper lobsters to develop feeds that are both nutritious and cost-effective, ensuring sustainable and profitable aquaculture practices. The study found that higher protein levels in the feed increase the lobsters’ growth performance and affect their body composition. It provides the first insights into the protein requirements of slipper lobsters and highlights that the most expensive high-protein feeds may not always be the best choice if the protein is not efficiently used for growth. Therefore, further research is needed to refine these feed formulations for optimal growth and nutrient utilization.

## 1. Introduction

The slipper lobster, *Thenus australiensis*, is a species of significant commercial value and a promising candidate for intensive aquaculture [1,2,3]. The recent achievement of completing its life cycle in captivity using only formulated feeds [3] has laid the foundation for developing sustainable aquaculture. The next critical step in establishing a successful aquaculture is developing a nutritionally adequate and economically viable formulated feed for all juvenile stages.

Building on the approach proposed by Carter and Codabaccus [4], and following the identification of suitable protein sources for *T. australiensis* [2,5], the subsequent critical step in advancing feed development is to enhance our understanding of the species’ protein nutrient requirements. Protein requirements in aquatic species are a pivotal focus of numerous nutritional studies, as dietary protein significantly impacts feed costs and is integral to various metabolic processes, including growth. Consequently, optimizing dietary protein is essential for achieving sustainable aquaculture [6].

Determining digestible protein (DP) requirements in conjunction with energy utilization provides broad nutritional requirements before delving into detailed investigations of individual amino acid needs [7,8,9]. Efficient protein assimilation into new tissues is affected by both the quality and quantity of available protein, as well as carbohydrates and lipids that supply non-protein energy [10,11,12]. Providing an optimal protein amount is crucial; insufficient dietary protein can lead to growth retardation, while excess dietary protein may result in increased ammonia-nitrogen excretion due to its inefficient use as an energy substrate. When formulated feeds lack sufficient non-protein energy sources, protein catabolism occurs to meet energy needs, thereby compromising somatic growth [13,14,15]. Consequently, an optimal feed formulation must balance protein and non-protein energy sources to meet energy requirements, thus maximizing dietary protein utilization for somatic growth [15,16]. More nutritional and physiological research is required to improve the retention efficiency from protein synthesis and enhance the dietary protein-sparing effects [6,11,17]. In contrast to many other aquaculture species, including various decapod crustaceans, there is limited information regarding the effect of dietary protein on *T. australiensis* nutritional physiology [2].

Optimal dietary protein content for crustaceans is 30 to 50% for some species of shrimps and lobsters and up to 60% for some post-larvae [18]. Previous studies on spiny lobsters showed variations in growth response to several protein and lipid levels. In general, for spiny lobsters, crude protein (CP) requirements appear to be higher for warm-water species in comparison to cold-water species. *Panulirus cygnus* showed high protein requirements (≥55%) [19], and *P. ornatus* grew the best at high protein levels (53–61%) and higher lipid content (10%) [20,21]. For *Jasus edwardsii*, maximum growth was achieved at dietary protein between 42 and 47% (DP: 33–35%) [9]. Until now, minimal research has focused on slipper lobster, and information on growth performance and nutrient requirements is limited [2,3,22]. However, good growth performance was achieved with formulated feeds containing around 65% crude protein (DP: 62%) and 17% total lipid on a dry matter basis [2].

Previous studies have established that different lobster species have different protein requirements. Hence, formulated feeds must be tailored for each species. Consequently, understanding the protein requirements of *T. australiensis* is vital for developing a well-balanced, cost-effective dietary formulation. Given that commercial protein ingredients typically contain 40–60% CP, the primary objective of the present study was to investigate dietary protein levels within this range. Such a foundation aids in creating benchmark feeds for future nutrient requirement research and contributes to nutrient requirement models.

## 2. Materials and Methods

### 2.1. Feed Manufacture

Experimental feeds (Table 1), adapted from Wirtz [2], were formulated to be isoenergetic with three CP levels: 45% (P45), 50% (P50) and 55% (P55). Each feed was prepared by accurately weighing the amount of fixed dry ingredients, including 0.1% of the inert digestibility marker yttrium oxide and 1% Spirulina powder (Paddymelon, The Melbourne Food Depot) mixed into a bowl. The filler components, such as corn starch and diatomaceous earth, were then separately added to the dry ingredient mix and blended with a kitchen mixer. Lipid ingredients (krill oil and lecithin) were slowly added to the dry ingredients and blended into a homogeneous semi-dry crumble. Warm water (45 °C) was added incrementally to achieve a target moisture content of 38%, forming a friable dough suitable for extrusion.

Feed strands were manufactured by cold extruding the dough to 0.7 mm and 1.2 mm diameters in equal parts using a pasta extruder (La Monferrina Dolly II). Each feed batch was processed twice through the extruder to ensure additional homogenization of the feed ingredients. The freshly extruded feed strands were set for 12 h at 4 °C [22]. After setting, the strands were cut into 10 mm pellets and stored in sealed, air-tight containers at 4 °C. Fresh feeds were prepared fortnightly, and a subsample of each batch (10.10 ± 0.03 g fresh weight) was sampled and stored at −20 °C for subsequent chemical analysis (Table 1).

### 2.2. Experimental Animals

Juvenile *T. australiensis* were reared from eggs at the University of Tasmania, Institute for Marine and Antarctic Studies (IMAS), following established protocols for tropical rock lobsters [24,25]. Post-hatchery, the juvenile slipper lobsters were initially cultured on fresh bivalve (*Plebidonax deltoides*) gonad until the third juvenile instar (J3). Subsequently, they were weaned onto a commercial-in-confidence nursery feed until reaching the intermolt J4 stage.

### 2.3. Experimental Design

Ninety-six lobsters, each with an average initial wet weight of 6.1 ± 0.3 g (1.3 ± 0.1 g dry weight (DW), mean ± S.E.) were randomly distributed across 12 experimental tanks (0.38 m length × 0.24 m width × 0.25 m height, 18 L) at a density of eight lobster per tank. An additional eight lobsters were measured and sampled at the time of stocking to ascertain the baseline chemical composition of the group. Four replicate tanks were assigned to each feed treatment, and the experiment was conducted for 74 consecutive days. The experimental tanks were supplied with filtered, fresh ozonated seawater at a rate of six exchanges h^−1^ and maintained under a 12:12 h blue light: dark photoperiod, set up as a flow-through system. Dissolved oxygen (107.9 ± 0.5% saturation), pH (8.2 ± 0.0), temperature (26.8 ± 0.0 °C), salinity (33.6 ± 0.1‰), and oxidation-reduction potential (339.8 ± 1.2 mV) were monitored daily, ensuring that the environmental conditions remain within the optimum range of this species. Experimental feeds were supplied at a feed rate of 2% animal body weight, in excess of daily requirements continuously over 18 h d^−1^ (approximately 15:00 to 09:00 h daily) by the even distribution of feed on belt feeders. The feed ration was adjusted by assessing mortalities and fortnightly bulk weight measurements. Mortalities and molting events were documented, and exuvia were promptly removed from the tank upon observation. Molting frequency was assessed by counting freshly shed exuviae twice daily at 8.00 a.m. and 4.00 p.m.

At the end of the experiment, all surviving lobsters (n = 65) were subjected to morphological measures of body weight (±0.001 g), carapace length (CL, ±0.01 mm) and carapace width (CW, ±0.01 mm). Two individuals per tank (n = 8 per treatment) were randomly selected and dissected to sample approximately 1 g of tail muscle tissue (TM) and to remove the hepatopancreas (HP). The remaining carcass, TM and HP samples were individually stored at −20 °C until chemical composition analysis.

Growth performance was evaluated by calculating the daily weight gain (WG, g DM d^−1^) and the specific growth rate (SGR, % BW d^−1^) using the following calculations [26,27,28]:

(1) $$Daily\;WG\;\left(g\;DM\;d^{-1}\right)=\frac{\left(final\;DW\;\left(g\right)-initial\;DW\;\left(g\right)\right)}{experimental\;duration\;(d)},$$

(2) $$SGR\;\left(\%\;BW\;d^{-1}\right)=\left[\frac{\left(ln\left(final\;DW\;(g)\right)-ln\left(initial\;DW\;(g)\right)\right)}{experimental\;duration\;(d)}\right]\times 100.$$

### 2.4. Apparent Feed Intake

Apparent feed intake (AFI) was measured daily [22,29]. Briefly, the uneaten feed was collected daily by siphoning into collectors equipped with a 150 µm mesh screen, rinsed with distilled water to remove salts and stored at −20 °C. The daily uneaten feed collection was cumulated for seven consecutive days and then averaged to represent the daily uneaten feed. The dry weight of the uneaten feed samples (n = 4 per experimental feed per week) was determined after oven drying at 105 °C for 24 h [30]. Upon termination of the experiment, feed DM loss due to leaching was determined for each experimental feed to correct feed intake. Dry matter loss was measured by providing the average experimental feed ration of 1.49 ± 0.06 g over three consecutive days under standard experimental conditions but without lobsters. Consistent with the daily experimental routine, feed pellets were siphoned into collectors equipped with a 150 µm mesh screen, rinsed with distilled water, dried for 24 h at 105 °C, and finally weighed. Apparent feed intake (AFI, g DM lobster^−1^ d^−1^), feed efficiency ratio (FERd, g DM g DM^−1^), protein efficiency ratio (PER), protein productive value (PPV, %) and energy productive value (EPV, %) were calculated as follows [5,31]:

(3) $$AFI\;\left(g\;DM\;{lobster}^{-1}\;d^{-1}\right)=\frac{\left(daily\;feed\;in\;\left(g\;DM\right)-daily\;feed\;out\;\left(g\;DM\right)\right)}{\left(1-feed\;proportional\;DM\;loss\right)}/number\;of\;lobster\;per\;tank,$$

(4) $$FERd\;(g\;DM\;{g\;DM}^{-1})=weight\;gain\;(g\;DM)/feed\;intake\;(g\;DM),$$

(5) PER=weight gain (g DM)/protein intake (g DM),

(6) $$PPV\;\left(\%\right)=protein\;gain\;\left(g\;DM\right)/protein\;intake\;\left(g\;DM\right)\times 100,$$

(7) $$EPV\;(\%)=energy\;gain\;\left(MJ\right)/energy\;intake\;\left(MJ\right)\times 100.$$

### 2.5. Apparent Digestibility

The apparent digestibility of experimental feed was assessed following the methodology described by Wirtz [2]. Briefly, each experimental feed included 0.1% yttrium oxide (Y_2_O_3_) as an indigestible marker. Fecal strands were collected within 2 h of egestion using a disposable pipette, placed onto a 250 μm screen and rinsed with distilled water to remove any salt. Daily fecal collections from a single experimental tank were pooled over a period of 74 days and stored at −20° C until chemical composition analyses. The apparent digestibility coefficient (ADC, %) of dry matter (DM), crude protein (CP), total lipid (TL) and gross energy (GE) was calculated according to [2,32]:

(8) $${ADC}_{DM}\;(\%)=(1-Y_{Feed}/Y_{Faeces})\times 100,$$

(9) $${ADC}_{N}\;\left(\%\right)=\left[1-\left(Y_{Feed}/Y_{Faeces}\right)\times \left({\%N}_{Faeces}/{\%N}_{Feed}\right)\right]\times 100,$$

where ADC_DM_ represents the ADC of DM in the feed; Y_Feed_ and Y_Faeces_ signify the proportion of the inert marker yttrium oxide in parts per million in the feed and faeces, respectively; ADC_N_ represents the ADC of nutrients CP, TL and GE; N_Feed_ and N_Faeces_ is the proportion (%) of CP, TL and GE in the feed and faeces, respectively.

### 2.6. Chemical Composition Analyses

All samples, including experimental feeds, animal tissues and feces, were freeze-dried (FD) to a constant weight. Subsequently, they were prepared for chemical composition analysis by grinding to a homogenous powder mechanically using an analytical mill (A11 basic Analytical mill, IKA^®^; IKA-Werke GmbH & Co. KG, Staufen, Germany) and manually using a mortar and pestle. The dry matter of FD samples was determined gravimetrically after oven drying at 105 °C for 24 h [30]. All chemical composition analyses were performed on FD samples and corrected for DM. Crude protein content was calculated by determining the elemental nitrogen (N) composition using flash combustion isotope ratio mass spectrometry (varioPYRO cube coupled to isoprime 100 mass spectrometer) and applying a conversion factor of 6.25 × N. The total lipid content was determined gravimetrically using a modified method from Bligh and Dyer [33], where total lipid was extracted in a mixture of dichloromethane, methanol, and milliQ water (1:1:0.9 *v*/*v*/*v*) according to Yagiz [34], substituting chloroform with dichloromethane [35]. Ash content was measured by combusting FD samples in a furnace at 600 °C for 2 h [36]. Carbohydrate content (g kg^−1^ DM basis) was calculated as; 100 − (crude protein + total lipid + ash). Energy content of the experimental feeds and whole-body samples was calculated using factors 23.9 MJ kg^−1^, 39.8 MJ kg^−1^ and 17.6 MJ kg^−1^ for protein, lipid and carbohydrate, respectively [23]. For tail muscle and hepatopancreas samples, only the factors for protein and lipid were applied.

Yttrium oxide was quantified as Y following acid digestion using nitric acid (70%, Merck KGaA, Darmstadt, Germany) and hydrogen peroxide (30%, Sigma-Aldrich Co., St. Louis, MO, USA). For AD measurements, approximately 100 ± 0.05 mg of FD feed and 50 ± 0.05 mg of FD faeces were digested overnight at room temperature with 2 mL of nitric acid, then heated to 100 °C for 2 h and left to cool. One mL of hydrogen peroxide was then added, and the mixture was reheated to 100 °C for 2 h. Digested samples were diluted to a volume of 10 mL with distilled water and analyzed using high-resolution inductively coupled plasma mass spectrometry (Thermo Scientific Element 2™ HR ICP-MS, Franklin, MA, USA) at the Central Science Laboratory, University of Tasmania, Australia.

### 2.7. Statistical Analyses

The mean values of replicate tanks (n = 4) are presented with their corresponding standard error of the mean (S.E.). Statistical analyses were conducted using RStudio (Version 1.2.5042, © 2009–2020 RStudio, Inc., Inc., Boston, MA, USA). Before analysis, percentage data underwent arcsine transformation. Normality and homogeneity of variances were assessed using the Shapiro–Wilk and Bartlett’s test, respectively. When assumptions were met, a one-way ANOVA and a Tukey’s HSD post hoc test were applied. For data that were non-normal or exhibited heteroscedasticity, the Kruskal–Wallis test (KWt) followed by a pairwise Wilcoxon rank-sum post hoc test was conducted [37]. Statistical significance was set at *p* < 0.05.

## 3. Results

### 3.1. Apparent Digestibility

Apparent digestibility was significantly affected by dietary protein (Table 2). A pairwise comparison indicated that the DM content of P45 and P50 were digested significantly less than that of P55. Crude protein digestibility was also affected by dietary protein, with P45 significantly less digestible than P50 and P55. All experimental feeds had significantly different energy digestibility, with ADC_GE_ increasing with increasing dietary protein content. Only ADC_TL_ was not significantly influenced by the dietary protein content.

### 3.2. Growth Performance

The initial lobsters were uniformly sized (21.9 ± 0.20 mm (CL) and 29.48 ± 0.26 mm (CW)) and weight (1.33 ± 0.03 gDW, *p* > 0.05) among treatments. Dietary protein levels did not significantly influence survival (68 ± 5%). Growth was evident in all treatments, and significant treatment effects (*p* < 0.05) were found for all final measurements and growth rates (Table 3). Increasing dietary protein levels significantly increased final dry weight and daily WG (*p* < 0.001). Total daily WG, SGR, CL and CW increment and molt frequency of juvenile lobsters in the P55 treatment were significantly higher than P45, with no significant differences between P50 compared to both P45 and P55.

Increasing dietary protein levels significantly increased AFI (Table 3), affecting dietary protein and energy intakes. As a result, lobsters in the P55 treatment gained the most protein and energy, significantly more than lobsters fed P50 and P45, with no significant differences between the lower dietary protein treatments. On a dry weight gain basis, the feed efficiency ratio was significantly higher in P45 than in P55, with no significant differences among the other treatments. Dietary protein levels significantly affect PER and PPV, with higher results in P45 than in P50 and P55. Dietary protein levels did not affect EPV; the average EPV was 0.29 ± 0.01.

### 3.3. Chemical Body Composition

Dietary protein levels significantly influenced the chemical composition of whole lobsters but not the tail muscle and hepatopancreas individually (Table 4). Lobsters fed P55 had significantly higher CP content than lobsters fed P50, but both did not differ significantly from lobsters in the P45 treatment. P55 lobsters had the highest TL and GE content, significantly higher than lobsters in the P45 and P50 treatments. The different dietary protein levels did not affect whole lobsters’ DM (22.5 ± 0.5%) and ash (38.2 ± 0.9%).

## 4. Discussion

Critical to developing a cost-effective formulated feed for any species is to establish a dietary protein level that the animal efficiently uses for optimum growth. It is a critical preliminary phase in developing feeds for new aquaculture species [4]. Optimizing the protein and energy content in aquaculture feed is the primary approach to enhance resource utilization efficiency in aquaculture and is crucial for effective protein deposition in the animals [6,7,38]. In nutrient partitioning, an optimal balance between digestible protein and energy assumes that the energetic cost of protein deposit is directly proportional to the amount of protein deposited. Such an assumption implies that absorbed amino acids are preferentially used for body protein synthesis rather than oxidative catabolism. Therefore, understanding the interaction between protein and energy is considered more important than knowledge of the individual nutrient requirements [7,9,39]. Consequently, the present study investigated dietary protein levels in formulated feeds for juvenile *T. australiensis* in relation to protein and energy efficiencies. This study provides a baseline to develop benchmark feed for this species that can contribute to future work on nutrient requirements, including requirement models.

### 4.1. Apparent Digestibility

Including corn starch as a bulking agent to achieve isoenergetic feeds with different dietary protein levels impacted feed digestibility for *T. australiensis* juveniles. Overall, ADC_DM_ in the present study was lower than previously reported for juvenile *T. australiensis* [2]. The reduced ADC_DM_ can be attributed to corn starch levels as the present study showed an inverse relationship between carbohydrate content and ADC_DM_. The higher the corn starch level, the lower the ADC_DM_. Previous studies have shown that carbohydrate sources significantly affect ADC_DM_ in crustaceans and fish with reduced digestibility by higher inclusion levels [40,41]. ADC_GE_ also showed an inverse relationship with the corn starch level, indicating that the lobster poorly digested energy from corn starch. Similarly, higher inclusion levels of gelatinized maize starch in feeds for *J. edwardsii* resulted in lower digestibility [40]. The lowest ADC_CP_ was measured in the experimental feed containing the lowest CP and the highest corn starch level, agreeing with previous studies on several fish species [41,42].

*Thenus australiensis* exhibits predominantly carnivorous feeding habits [43,44], targeting a diverse array of prey, including gastropods, bivalves, chitons, crustaceans, sea urchins, polychaetas, and occasionally fish [45,46,47]. Carnivorous crustaceans have a limited ability to utilize carbohydrates [48] and the trend of decreased digestibility with increasing starch levels is thought to be due to the saturation of digestive carbohydrate enzymes by the substrate [41]. The optimal carbohydrate content for slipper lobster feed is not well-documented in scientific literature, highlighting the need for further research.

### 4.2. Growth Performance

The survival rate observed in the present study aligns with previous research on the nutrition of juvenile *T. australiensis*, which reported survival rates ranging from 66.7% to 100.0% [5,22,31]. Growth performance of the present study was comparable with previous studies [5,31], which used higher amounts of dietary protein (598–651 g kg^−1^) and an average protein/energy ratio of 27.4 ± 0.5 g CP MJ GE^−1^. The effect of dietary protein and energy levels is studied on several lobster and prawn species and usually demonstrates that with the application of different isoenergetic feeds, growth parameters such as WG and SGR have a positive relationship with increasing protein-to-energy ratio, while other parameters like FER, PER and PPV had a negative correlation [49,50,51]. The present study confirms that increasing dietary protein levels significantly enhance growth parameters while inversely affecting FERd, PER, and PPV.

Previous studies have established that lobster grew best when fed high protein feeds, with growth increasing linearly with increasing protein content. Juvenile *P. ornatus* [21] and *P. cygnus* [19] exhibited the highest growth at the highest protein content of 612 g CP kg^−1^ (563 g DP kg^−1^) and 506 g CP kg^−1^, respectively, with no evidence of diminishing growth responses at higher dietary protein levels. A similar finding was observed in the present study as juvenile *T. australiensis* growth performance increased with increasing dietary protein levels with no indication of reduced growth at the highest dietary protein level of 550.9 g CP (518.2 g DP kg^−1^). However, in earlier studies, higher dietary CP levels (600–650 g kg^−1^) resulted in comparable growth performance to the highest protein level from this study, suggesting a plateau in growth beyond 55% CP for *T. australiensis*. For juvenile *J. edwardsii*, there appears to be a clear dietary protein optimum at 29 and 31% digestible protein when lobster feeds contain 5% and 9% lipid, respectively [9].

In the present study, the feed producing the highest growth performance (P55) contained 17.2 MJ kg^−1^ of digestible energy, equating to a DP/DE ratio of 30.1 g MJ^−1^. Experimental results suggest that the optimal DP/DE ratio has not been demonstrated. However, the DP/DE ratios used in the present study were in the range previously described as optimal for other lobster species. The optimal DP/DE ratio for juvenile *J. edwardsii* for maximum weight gain was 29 g DP MJ DE^−1^ [9], similar to those reported for *P. ornatus* (29.8 g DP MJ^−1^) [20] with both lobsters displaying decreased weight gain beyond the optimal digestible DP/DE ratio.

PER decreased with increased dietary protein levels, and PPV was the highest in P45, which agrees with previous findings [15,50], indicating that *T. australiensis* tend to conserve and utilize protein for growth at lower dietary protein levels, while a higher proportion of dietary protein in P50 and P55 might have also been used to generate energy. When growth rates are high, protein retention efficiency directly measures how effectively nutrients are utilized for tissue growth. In the present study, PPV ranged from 29 to 45%, which is higher than the 7.4–19.0% recorded for *J. edwardsii* [9,52]. Furthermore, with the highest corn starch and carbohydrate content, P45 provided an additional non-protein energy source, allowing the ingested protein to be utilized more efficiently for growth. In *P. monodon*, carbohydrates have been shown to spare protein more efficiently than lipids [53,54], and it also appears that carbohydrates are more important in the diet of small lobster [21].

### 4.3. Chemical Body Composition

Chemical whole-body and tissue compositions are comparable with those previously described for juvenile *T. australiensis* [31] with slight deviations, which can be traced back to different experimental feeds and research aims. The present study used isoenergetic and isolipidic experimental feeds; therefore, variation in nutrient and energy accumulation can be attributed to the dietary protein and carbohydrate (corn starch) levels rather than energy and lipid content. Dietary protein levels did not significantly affect the chemical composition of *T. australiensis* tissues individually (TM and HP). Still, whole-body samples presented significant differences in protein, lipid, and energy. In oriental river prawns (*Macrobrachium nipponense*), it was observed that the mid dietary protein level treatment (369.5 g CP kg^−1^) showed the lowest protein composition (659.3 g CP kg^−1^) [50]. Similar was found in the present study, where the whole-body protein content of P50 lobsters was the lowest, significantly lower than P55. The lowest lipid content of P50 may have resulted in the utilization of protein as the primary energy source, leading to reduced protein deposition. In contrast to the present study, lipid content in the whole-body *M. nipponense* significantly decreased when the dietary protein levels increased [50]. In the present study, the higher whole-body lipid content of P55 may indicate that protein and carbohydrates (corn starch) were preferentially metabolized to meet general energy requirements and that the excess lipid was stored rather than used for energy.

The present study provided the first insight into the protein nutrition of this species and the first step toward practical improvements in feed formulations [4], emphasizing the importance of growth, feed efficiencies, and feed cost considerations. In a commercial context, higher growth performance on a given feed may not necessarily be the best practice or the sole criterion for successful operation, but rather the cost implications associated with this higher growth. Protein is an expensive nutrient [9,55]; therefore, high-protein feeds are more costly. Consequently, if the protein in a high-protein feed is not utilized efficiently for growth, then, commercially, it may be economically beneficial to target a lower-protein feed (economical dietary protein level, [56]). The goal is to develop feed formulations that are nutritionally adequate and economically viable, ensuring sustainable and profitable aquaculture practices. This holistic approach to feed formulation can help achieve long-term success in industry.

## 5. Conclusions

In conclusion, this study revealed that dietary protein levels significantly influence the growth rate of juvenile *T. australiensis*, with the highest growth rate achieved at 550.9 g CP kg^−1^ (518.2 g DP kg^−1^). This research provides the first comprehensive insight into the protein nutrition of this species and represents an initial step toward practical improvements in feed formulations. Nonetheless, given the observed disparity between high growth performance and protein utilization, further research is essential to refine these feed formulations to achieve high growth coherence with high protein efficiency.

## Figures and Tables

**Table 1:** Formulation and chemical composition of homogenized composite experimental feeds with three dietary protein inclusion levels.

Ingredient	Experimental Feeds		
	P45	P50	P55
Ingredients (g kg^−1^ as-is)			
Basal mix ^1^	701	766	831
Corn starch	244	164	83
Diatomaceous earth	44	59	75
Spirulina ^2^	10	10	10
Yttrium oxide	1	1	1
Total	1000	1000	1000
Chemical composition (g kg^−1^ DM)			
Dry matter	951	949	951
Crude protein	445	490	551
Total lipid	75	65	70
Ash	107	122	143
Carbohydrate ^3^	373	323	236
Gross energy (MJ Kg^−1^) ^4^	20	20	20
CP:GE (g CP MJ GE) ^5^	22	25	28
Digestible composition (g kg^−1^ DM)			
Dry matter	720	734	757
Protein	375	458	518
Lipid	49	38	42
Energy (MJ Kg^−1^)	16	17	17
DP:DE (g DP MJ DE^−1^) ^6^	24	28	30

Experimental feeds were subsampled fortnightly (n = 5 per feed) to reflect the average feed composition over the experimental phase. ^1^ Commercial in confidence basal mix. ^2^ Spirulina powder, Paddymelon, The Melbourne Food Depot. ^3^ Carbohydrate = 100 − (crude protein + total lipid + ash). ^4^ Calculated by using factors 23.9 MJ kg^−1^, 39.8 MJ kg^−1^ and 17.6 MJ kg^−1^ for proteins, lipids and carbohydrates, respectively [23]. ^5^ Crude protein/gross energy ratio. ^6^ Digestible crude protein/digestible energy ratio.

**Table 2:** Apparent digestibility coefficients (%) for dry matter (ADC_DM_), crude protein (ADC_CP_), total lipid (ADC_TL_) and gross energy (ADC_GE_) in juvenile *Thenus australiensis* fed experimental feeds with three different dietary protein levels (mean ± S.E., n = 4).

	Experimental Feeds			Statistics	
	P45	P50	P55	F	*p*
*ADC_DM_*	75.7 ± 0.4 ^a^	77.4 ± 0.7 ^a^	79.6 ± 0.3 ^b^	16.65	0.001
*ADC_CP_*	84.2 ± 2.3 ^a^	93.5 ± 0.6 ^b^	94.1 ± 1.6 ^b^	10.03	0.009
*ADC_TL_*	63.8 ± 2.0	57.8 ± 1.4	59.3 ± 1.8	2.98	0.108
*ADC_GE_*	79.2 ± 0.5 ^a^	83.0 ± 0.3 ^b^	86.2 ± 0.5 ^c^	62.37	<0.001

Significant differences (*p* < 0.05), determined by Tukey’s HSD post hoc test, are donated with letters in superscript (a–c).

**Table 3:** Growth performance data (mean ± S.E., n = 4) of juvenile *Thenus australiensis* grown on experimental feeds with three crude protein inclusion levels after 74 days.

	Experimental Feeds			Statistics		
	P45	P50	P55	Test	F or χ^2^	*p*
Initial weight (g DM)	1.35 ± 0.13	1.35 ± 0.12	1.27 ± 0.11	ANOVA	0.58	0.582
Final weight (g DM)	4.57 ± 0.37 ^a^	5.27 ± 0.35 ^b^	5.89 ± 0.33 ^c^	ANOVA	19.89	<0.001
Daily WG (g DM d^−1^)	0.04 ± 0.00 ^a^	0.05 ± 0.00 ^b^	0.06 ± 0.00 ^c^	ANOVA	20.12	<0.001
SGR (% dry BW d^−1^)	1.63 ± 0.09 ^a^	1.84 ± 0.04 ^ab^	2.07 ± 0.08 ^b^	ANOVA	9.44	0.007
CL increment (mm)	10.76 ± 0.33 ^a^	12.98 ± 0.76 ^ab^	13.87 ± 0.77 ^b^	ANOVA	6.1	0.021
CW increment (mm)	15.10 ± 0.46 ^a^	17.67 ± 1.07 ^ab^	18.92 ± 0.89 ^b^	ANOVA	5.26	0.031
Survival (%)	71.88 ± 10.67	59.38 ± 7.86	71.88 ± 7.86	ANOVA	0.69	0.525
Molt frequency (molts lobster^−1^)	2.02 ± 0.03 ^a^	2.11 ± 0.17 ^ab^	2.71 ± 0.10 ^b^	KWt	6.73	0.035
AFI (g DM lobster^−1^ d^−1^)	0.10 ± 0.00 ^a^	0.15 ± 0.00 ^b^	0.19 ± 0.01 ^c^	ANOVA	74.83	<0.001
FERd (g DM g DM^−1^)	0.45 ± 0.03 ^b^	0.37 ± 0.01 ^ab^	0.34 ± 0.03 ^a^	ANOVA	6.41	0.019
Protein intake (g) ^1^	3.10 ± 0.08 ^a^	5.23 ± 0.12 ^b^	7.54 ± 0.33 ^c^	ANOVA	113.1	<0.001
Protein gain (g) ^2^	1.40 ± 0.10 ^a^	1.50 ± 0.08 ^a^	2.21 ± 0.12 ^b^	ANOVA	19.94	<0.001
PER	1.02 ± 0.06 ^b^	0.75 ± 0.03 ^a^	0.62 ± 0.05 ^a^	ANOVA	18.38	<0.001
PPV (%)	45.18 ± 3.75 ^b^	28.72 ± 1.41 ^a^	30.07 ± 1.51 ^a^	ANOVA	13.79	0.002
Energy intake (MJ) ^1^	0.14 ± 0.00 ^a^	0.21 ± 0.00 ^b^	0.27 ± 0.01 ^c^	ANOVA	73.91	<0.001
Energy gain (MJ) ^2^	0.05 ± 0.00 ^a^	0.06 ± 0.00 ^a^	0.08 ± 0.00 ^b^	ANOVA	25.57	<0.001
EPV (%)	33 ± 3	26 ± 1	28 ± 1	ANOVA	2.89	0.107

WG: weight gain; SGR: specific growth rate; CL: carapace length; CW: carapace width; AFI: Apparent feed intake; FERd: Feed efficiency ratio; PER: protein efficiency ratio; PPV: Protein productive value; EPV: Energy productivity value; KWt: Kruskal–Wallis test. Significant differences (*p* < 0.05), determined by Tukey’s HSD post hoc or pairwise Wilcoxon rank-sum post hoc test, are donated with letters in superscript (a–c). ^1^ Protein and energy intakes were calculated from experimental feed content and apparent feed intake. ^2^ Protein and energy gains were assessed by comparing the initial and final protein and energy content of the lobsters.

**Table 4:** Whole body and tissue chemical composition and gross energy content in juvenile *Thenus australiensis* fed experimental feeds with different dietary protein levels over a 74-day growth experiment (mean ± S.E., n = 4).

	Experimental Feeds			Statistics	
	P45	P50	P55	F	*p*
Whole-body					
Dry matter (% WW)	22.3 ± 1.1	21.9 ± 1.2	23.4 ± 0.8	0.866	0.453
Crude protein (% DM)	42.5 ± 1.2 ^ab^	38.4 ± 1.3 ^a^	46.0 ± 2.1 ^b^	5.606	0.026
Total lipid (% DM)	2.8 ± 0.4 ^a^	2.8 ± 0.4 ^a^	6.1 ± 1.0 ^b^	7.453	0.012
Ash (% DM)	40.1 ± 1.7	38.5 ± 1.3	36.1 ± 0.8	2.341	0.152
Gross energy (MJ kg^−1^ DM)	13.8 ± 0.4 ^a^	13.9 ± 0.3 ^a^	15.5 ± 0.5 ^b^	5.557	0.027
Tail muscle					
Dry matter (% WW)	18.3 ± 0.8	19.0 ± 0.9	19.7 ± 0.5	0.915	0.435
Crude protein (% DM)	90.4 ± 1.8	87.6 ± 2.1	89.8 ± 2.3	0.467	0.641
Total lipid (% DM)	4.3 ± 1.5	7.6 ± 0.4	7.3 ± 0.6	3.521	0.074
Ash (% DM)	15.0 ± 1.4	13.6 ± 1.2	11.8 ± 0.7	1.948	0.198
Gross energy (MJ kg^−1^ DM)	23.3 ± 0.7	23.9 ± 0.5	24.4 ± 0.5	0.732	0.508
Hepatopancreas					
Dry matter (% WW)	21.3 ± 2.1	23.0 ± 3.6	25.6 ± 1.2	1.478	0.279
Crude protein (% DM)	46.7 ± 3.4	37.7 ± 4.6	41.0 ± 0.9	1.819	0.217
Total lipid (% DM)	44.0 ± 2.0	50.1 ± 7.7	49.6 ± 2.7	0.533	0.604
Ash (% DM)	7.5 ± 0.9	9.3 ± 1.7	9.9 ± 0.0	1.249	0.332
Gross energy (MJ kg^−1^ DM)	28.7 ± 1.1	29.0 ± 2.0	29.5 ± 0.9	0.096	0.909

Significant differences (*p* < 0.05), determined by Tukey’s HSD post hoc test, are denoted with letters in superscript (a,b). WW: wet weight; DM: dry matter.

## Data Availability

The presented data are available on request from the first authors.
